# Rational design of deep eutectic solvents for the stabilization of dehydrogenases: an artificial neural network prediction approach

**DOI:** 10.3389/fchem.2024.1436049

**Published:** 2024-08-01

**Authors:** Mia Radović, Ana Jurinjak Tušek, Tamara Reiter, Wolfgang Kroutil, Marina Cvjetko Bubalo, Ivana Radojčić Redovniković

**Affiliations:** ^1^ Faculty of Food technology and Biotechnology, University of Zagreb, Zagreb, Croatia; ^2^ Institute of Chemistry, University of Graz, Field of Excellence BioHealth, BioTechMed Graz, Graz, Austria

**Keywords:** COSMO-RS, deep eutectic solvents, dehydrogenases, enzymatic stability, neural networks, rational design

## Abstract

Stabilized enzymes are crucial for the industrial application of biocatalysis due to their enhanced operational stability, which leads to prolonged enzyme activity, cost-efficiency and consequently scalability of biocatalytic processes. Over the past decade, numerous studies have demonstrated that deep eutectic solvents (DES) are excellent enzyme stabilizers. However, the search for an optimal DES has primarily relied on trial-and-error methods, lacking systematic exploration of DES structure-activity relationships. Therefore, this study aims to rationally design DES to stabilize various dehydrogenases through extensive experimental screening, followed by the development of a straightforward and reliable mathematical model to predict the efficacy of DES in enzyme stabilization. A total of 28 DES were tested for their ability to stabilize three dehydrogenases at 30°C: (*S*)-alcohol dehydrogenase from *Rhodococcus ruber* (ADH-A), (*R*)-alcohol dehydrogenase from *Lactobacillus kefir* (Lk-ADH) and glucose dehydrogenase from *Bacillus megaterium* (GDH). The residual activity of these enzymes in the presence of DES was quantified using first-order kinetic models. The screening revealed that DES based on polyols serve as promising stabilizing environments for the three tested dehydrogenases, particularly for the enzymes Lk-ADH and GDH, which are intrinsically unstable in aqueous environments. In glycerol-based DES, increases in enzyme half-life of up to 175-fold for Lk-ADH and 60-fold for GDH were observed compared to reference buffers. Furthermore, to establish the relationship between the enzyme inactivation rate constants and DES descriptors generated by the Conductor-like Screening Model for Real Solvents, artificial neural network models were developed. The models for ADH-A and GDH showed high efficiency and reliability (R^2^ > 0.75) for *in silico* screening of the enzyme inactivation rate constants based on DES descriptors. In conclusion, these results highlight the significant potential of the integrated experimental and *in silico* approach for the rational design of DES tailored to stabilize enzymes.

## 1 Introduction

Deep Eutectic Solvents (DES), as innovative and tunable liquid media, are gaining attention for their non-toxic, versatile properties applicable across diverse domains such as synthesis, catalysis, separation processes, material science and biomedicine (Zhang et al., 2012). Originally coined to describe mixtures solidifying at temperatures lower than individual components’ crystallization points, DES now encompass combinations maintaining a liquid state at specific temperatures (Abranches and Coutinho, 2023). Deep eutectic solvents (DES) are commonly prepared by mixing two or more components, like a hydrogen bond donor (HBD) and a hydrogen bond acceptor (HBA), in precise molar ratios to achieve a liquid state at ambient or application-specific temperature. DES formulations may incorporate various compounds such as carboxylic acids, sugars and polyols as HBDs, and metal chlorides, metal oxides and organic compounds (e.g., quaternary ammonium compounds) as HBAs. The choice of components is dictated by the intended properties and applications of the resulting DES. Water is often included as an additional component to reduce the melting point and viscosity of the mixture, as well as to enhance the DES’s effectiveness for specific uses (Zhang et al., 2012).

Utilizing the synergy of DES and biocatalysis as a biotechnological approach aligns seamlessly with the goal of achieving efficient and sustainable production of various commercially significant products. Biocatalysis facilitates complex transformations with high levels of regio-, chemo- and enantioselectivity under mild and cost-effective conditions. DES, in turn, offer robust support for modulating and directing reaction pathways towards desired products in an environmentally friendly manner (Panić et al., 2021). Given the vast array of structural possibilities inherent in DES, it becomes feasible to tailor an optimal DES for each specific enzymatic reaction system. This versatility enables DES to enhance substrate solubility/loading, enzyme activity and stability, increase reaction yields, modify biocatalyst stereopreference and contribute to the overall eco-friendliness of the process, including options for recycling and reuse. In the realm of (bio)catalytic processes, DES do not serve just as solvents or co-solvents, but also as smart co-substrates, enzyme storage media, extractive reagents for enzymatic products and pretreatment solvents for enzymatic biomass (Juneidi et al., 2017; Pätzold et al., 2019; Panić et al., 2021). Until 2020, hydrolases received the majority of attention in DES environments. However, in recent years, research has broadened to include other hydrolytic enzymes (such as epoxide hydrolases, phospholipases, proteases and haloalkane dehalogenase), as well as lyases and dehydrogenases (Panić et al., 2021; Zhang et al., 2024). Among the listed enzymes, alcohol dehydrogenases as vital enzymes facilitating reversible redox reactions to yield specific alcohols or ketones have been the focus of research by several research groups. Bittner et al. highlighted that choline chloride-based DES with high glycerol content are excellent stabilizers of horse liver alcohol dehydrogenase (HLADH), while Gajardo-Parra et al. demonstrated that DES based on glycerol and sorbitol enhance formate dehydrogenase (FDH) stability against thermal stress (Bittner et al., 2022; Gajardo-Parra et al., 2023). Most studies indicate that enzyme activity decreases in DES, especially in neat DES or those with low water content. For instance, Huang et al. reported that a minimum water activity of 0.2 was necessary for HLADH catalytic activity in choline chloride:glycerol/water mixtures (Huang et al., 2020). This finding was supported by Bittner et al. through molecular dynamics simulations, revealing a rigid HLADH structure in low-water DES (Bittner et al., 2022).

The quest for identifying an optimal DES for specific applications has traditionally relied on trial-and-error methods, lacking systematic exploration of DES structure-activity relationships. Consequently, the strategic design of these solvents remains relatively undeveloped. To advance their industrial utility, it's crucial to gather data on DES properties and develop mathematical tools for solvent design. The Conductor-like Screening Model for Real Solvents (COSMO-RS) offers a computational approach for generating molecular σ-profiles. These molecular descriptors provide essential information on a molecule’s electrostatic, hydrogen bonding and dispersion interactions, allowing for quantification of structural changes (Klamt et al., 1998a). Past studies have showcased the effectiveness, reliability and cost-efficiency of employing molecular descriptors generated by COSMO-RS in Quantitative Structure-Property Relationships (QSPR) and machine learning (ML) to predict physicochemical properties of DESs, such as density (Lemaoui et al., 2020b), viscosity (Benguerba et al., 2019), conductivity (Lemaoui et al., 2020a), melting point (García et al., 2015) and pH value (Panić et al., 2022). Additionally, in the context of DES applications, QSPR models for predicting the solubilities of gases and drugs have recently been successfully developed (Wang et al., 2021; Asghar et al., 2023). These models leverage the structural properties of various inorganic and organic molecules to forecast their behavior in DES, providing valuable insights for their application in various industrial processes. However, to the best of our knowledge, no QSPR models have been reported for predicting the behavior of complex biomolecules, such as enzymes, in these solvents. Developing predictive models could significantly enhance our ability to design and optimize bioprocesses in DES. Building upon the aforementioned, this study aimed to rationally design DES for the stabilization of various dehydrogenases through extensive experimental screening, followed by the development of a straightforward and reliable mathematical model to predict the efficacy of DES in enzyme stabilization. To achieve this, we initially experimentally tested the stabilization effects of 28 DES on three different dehydrogenases: (*S*)-alcohol dehydrogenase from *Rhodococcus ruber* (ADH-A), (*R*)-alcohol dehydrogenase from *Lactobacillus kefir* (Lk-ADH) and glucose dehydrogenase from *Bacillus megaterium* (GDH). Based on the calculated enzyme inactivation rate constants and DES descriptors generated by COSMO-RS, QSPR models for predicting the efficacy of DES in enzyme stabilization were developed using artificial neural networks (ANNs).

## 2 Materials and methods

### 2.1 Chemicals

Betaine (>99%), choline chloride (>98%), ethylene glycol (anhydrous, >99.8%), urea (>98%) and D-glucose (>99.5%) were purchased from Sigma Aldrich. NAD^+^ and propylene glycol were obtained from BASF while glycerol (>99%) was from Alfa Aesar. The HPLC grade 2-propanol was obtained from Honeywell.

### 2.2 Tested enzymes

For this research, cell-free extracts of three dehydrogenases were produced and investigated. Glucose dehydrogenase *Bacillus megaterium* (GDH, internal plasmid number pEG521) (Lampel et al., 1986) and two NAD(P)H-dependent alcohol dehydrogenases (ADHs), namely, ADH-A from *R. ruber* DSM 44541 (pEG10) (Karabec et al., 2010) and Lk-ADH from *L. kefir* (pEG326). Additional data provided in the Supplementary Material.

### 2.3 Solvent preparation

DES preparation was performed by mixing hydrogen bond acceptor (HBA), hydrogen bond donor (HBD) and water in defined molar ratios (Table 1). Before use, choline chloride was dried in the vacuum concentrator at 60°C for 24 h. Weighted HBA, HBD and water were placed in a glass container where the mixture was stirred and heated up to 60°C until a colourless and homogeneous liquid was formed. All individually prepared DES were stored at room temperature in sealed glass bottles and later used for stability experiments. For each enzyme, a reference buffer was chosen according to its optimal pH value and prepared according to standard protocols. More precisely, 50 mM TRIS-HCl buffer (pH = 7.5) was chosen for ADH-A and Lk-ADH, while 50 mM potassium-phosphate buffer (pH = 7.5) for GDH.

**TABLE 1:** List of DES used for ADHs activity and stability screening.

			Abbrev. (HBA:HBD)	Molar ratio of components	Water content (wt%)		
					10%	30%	50%
Betaine-based DES		1	B:EG	1:2	DES 1.1	DES 1.2	DES 1.3
		2	B:PG	1:3	DES 2.1	DES 2.2	DES 2.3
		3	B:Gly	1:2	DES 3.1	DES 3.2	DES 3.3
		4	B:U	1:3	x*	x*	DES 4.3
Choline chloride-based DES		5	ChCl:U	1:2	DES 5.1	DES 5.2	DES 5.3
		6	ChCl:U:EG	1:2:2	DES 6.1	DES 6.2	DES 6.3
		7	ChCl:U:Gly	1:2:2	DES 7.1	DES 7.2	DES 7.3
		8	ChCl:EG	1:2	DES 8.1	DES 8.2	DES 8.3
		9	ChCl:Gly	1:2	DES 9.1	DES 9.2	DES 9.3
		10	ChCl:PG	2:3	DES 10.1	DES 10.2	DES 10.3

Abbreviations: betaine (B), choline chloride (ChCl), ethylene glycol (EG), glycerol (Gly), propylene glycol (PG), urea (U). *B:U at molar ratio 1:3 with 10 and 30 wt% of water is a solid at room temp.

### 2.4 Model enzymatic activity assays

To ensure quick activity screenings of a large number of samples, spectroscopic enzymatic activity assays in 96-multi well plates were developed. For each enzyme, a model oxidation reaction was chosen. The common NADH formation rate was immediately monitored for 10 min at a fixed wavelength of 340 nm on a UV-Vis spectrophotometer (plate reader). Dehydrogenase activity (*A*, μmol min^-1^ dm^-3^) was calculated according to the expression:

$$A=\frac{\Delta A}{\Delta t}\cdot \frac{10^{6}}{\varepsilon_{340}\cdot d}$$

where *ΔA*/*Δt* is the absorbance change through time (min^-1^); *ε*_340_ is the extinction coefficient (6,220 cm^2^ mmol^-1^ for NADH at *λ* = 340 nm); *d* is cuvette diameter (0.61 cm for a well in a 96-well plate); 10^6^ is a conversion factor (cm^3^ μmol dm^-3^ mmol^-1^).

To ascertain whether the enzymes oxidize not only the substrates utilized in the assays but also the components of the deep eutectic solvents (DES), namely, polyols, we replaced the model substrates with pure DES components. Subsequently, the assays were conducted as described below.

#### 2.4.1 GDH activity assay

GDH activity assay was conducted directly in a 96-well plate with a total volume of 200 µL by adding 0.1 mg mL^-1^ of the enzyme (10 µL), 20 mM NAD^+^ (5 µL), 50 mM potassium-phosphate buffer pH = 7.5 (175 µL) and 200 mM glucose (10 µL) in that exact order. Upon mixing, the microplate was immediately measured on the plate reader.

#### 2.4.2 ADHs activity assay (ADH-A and Lk-ADH)

ADH-A and Lk-ADH activity assays were conducted directly in a 96-well plate with a total volume of 200 µL. 1 mg mL^-1^ of the enzyme (10 µL), 20 mM NAD^+^ (5 µL), 50 mM TRIS-HCl buffer pH = 7.5 (180 µL) and pure 2-propanol (5 µL) were added in that exact order, mixed and immediately measured on the plate reader.

### 2.5 Monitoring enzymatic stability

Stock solutions enzymes (0.1 mg mL^-1^ for GDH; 1 mg mL^-1^ for ADH-A and Lk-ADH) were separately prepared in 29 different solvents, namely, 28 aqueous DES and corresponding reference buffer (Table 1). A total of 29 individual stock solutions for each enzyme were kept in sealed glass vials in the dark and incubated at 30°C. Aliquots were periodically withdrawn from these vials, for stability measurements according to previously defined model enzymatic activity assays. For simultaneous evaluation of all stock solutions, experiments were performed in a 96-well plate and each measurement was carried out in triplicates. More precisely, each well represented an individual model oxidation reaction catalyzed by enzyme incubated in different solvents. For each measurement in time, a new 96-multi well plate was used.

Initially, activity measurements were conducted daily for 1 week, followed by measurements every 7 days until a 50% decrease in residual activity was observed. Furthermore, to capture any initial enzymatic activity changes, activity assessments on the first day of incubation were performed at 0, 2, 4, 6 and 8 h. Residual enzymatic activity (*A*_*R*_, %) was separately calculated for enzymes in each tested solvent and expressed as a percentage according to 100% activity measured in each tested solvent 20 min after preparing its stock solution (set as time zero). The decrease of residual enzymatic activity through time was described by the first-order kinetic model:

$$A_{R}\left(t\right)=A_{R,0}\cdot e^{-k\cdot t}$$

where *k* is the first-order rate constant (day^-1^) and *A*_*R*_ is residual enzymatic activity (either at time zero or at time *t)*. Kinetic parameters were estimated by fitting the experimental data to the nonlinear equation using the Levenberg–Marquardt algorithm implemented in WR Mathematica 10.0.

Half-life of enzymes (*t*_*1/2*_, day) was calculated based on determined *k*, the first-order rate constant (day^-1^), according to the equation for first-order reactions:

$$t_{1/2}=\frac{\ln2}{k}$$

### 2.6 DES σ-decriptors

**FIGURE 1:**
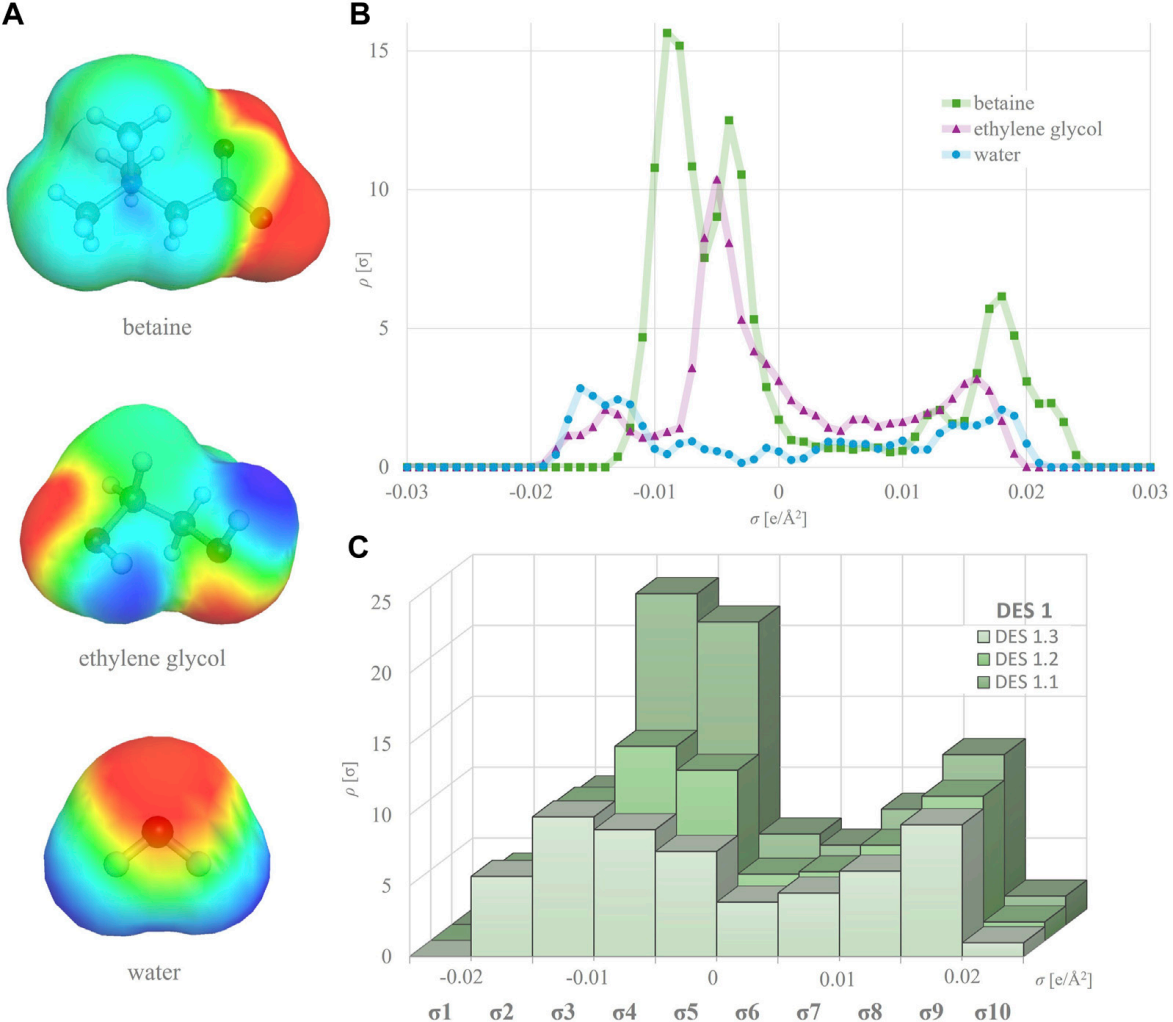
σ-surfaces of different DES components **(A)** and their individual σ-profiles **(B)** used to calculate σ-profiles of respective DES at different water shares **(C)**.

All DES constituents, including HBA, HBD and water molecules, underwent geometry and energy optimization using the BIOVIA TmoleX19 version 2021 (Dassault Systèmes) software. For quantum chemical calculations, DFT (density functional theory) with the BP86 functional level of theory and def-TZVP basis set were employed (Klamt et al., 1998b). To obtain a simplified and user-friendly database, only the single most abundant non-ionized conformer with the lowest energy was selected for each molecule and utilized for subsequent calculations. Molecules consisting of two or more ions (e.g., choline chloride) were treated as ion pairs (Abranches et al., 2019). The COSMO file for each optimized molecule, generated using BOVIA COSMOtherm version 2021 (Dassault Systèmes) software, contained its σ-profile curve (Figure 1B). Molecular descriptors for all DES constituents were defined based on their σ-profile curves, which were divided into 10 regions with a width of 0.005 e/Å^2^, covering the range from −0.025 to +0.025 e/Å^2^. The areas under the curve were integrated separately for each defined region. The ordinate values on the boundaries of the regions were evenly split and attributed to neighboring regions. Consequently, 10 S descriptors (S^1^–S^10^) of the σ-profiles were calculated, representing the numerical values of these 10 areas (Supplementary Table S1; Figure 1C). To generate a unique descriptor set for each specific DES, the σ-profiles of its constituents underwent the following processing. The descriptors of the studied DESs (S^i^_mix_) were derived from the HBA, HBD and water descriptors according to equation (Benguerba et al., 2019):

$$S_{\text{mix}}^{i}=\sum_{j=1}^{NC}X_{j}S_{\sigma -profile,j}^{i}$$

where *i* is the descriptor number (1–10), *j* is the DES constituent number, *X*_*j*_ is the molar fraction of HBA, HBD or water, S^i^_σ-profile,*j*_ is the *j*th constituent *i*th descriptor and *NC* is the total number of constituents from which DES is prepared.

### 2.7 Artificial neural network (ANN) modelling of correlation between enzymes’ inactivation rate constant and DES descriptors

It was assumed that the enzymes’ inactivation rate constant can be described as a function of the σ-profile of the mixture, expressed by a set of S^i^_mix_ descriptors according to equation:

$$k_{1}=f\left(S_{\text{mix}}^{1},S_{\text{mix}}^{2},S_{\text{mix}}^{3},S_{\text{mix}}^{4},S_{\text{mix}}^{5},S_{\text{mix}}^{6},S_{\text{mix}}^{7},S_{\text{mix}}^{8},S_{\text{mix}}^{9},S_{\text{mix}}^{10}\right)$$

Multilayer perceptron (MLP) artificial neural networks (ANNs) were used for the prediction of the enzymes’ inactivation rate constant based on S^i^_mix_ descriptors. The ANN input layer was different for individual enzymes; (i) for ADH-A input variables were S^3^_mix_, S^4^_mix_, S^5^_mix_, S^6^_mix_, S^7^_mix_, S^8^_mix_, S^9^_mix_ and S^10^_mix_; (ii) for GDH input variables were S^1^_mix_, S^5^_mix_, S^6^_mix_, S^7^_mix_, S^8^_mix_ and S^10^_mix_; (iii) for Lk-ADH input variables were S^1^_mix_, S^6^_mix_ and S^5^_mix_.The number of input variables was estimated based on the Spearman correlation matrix between enzymes’ inactivation rate constant and S^i^_mix_ descriptors. The number of neurons in the hidden layer varied between 4–13 and was randomly selected by the algorithm. The hidden activation function and output activation function were selected randomly from Identity, Logistic, Hyperbolic tangent and Exponential function. 84 experiment data were randomly divided into a calibration set (55 data points) and a prediction set (23 data points). The calibration data set was furthermore divided into 70% for network training, 15% for network testing and 15% for model validation. Model training was carried out using a back error propagation algorithm and the error function was a sum of squares implemented into Statistica v.14.0. The developed model’s performance was estimated by calculating R^2^ and Root Mean Squared Error (RMSE) values for the training, test and validation sets. The prediction performance of the models was estimated based on the coefficient of determination for prediction (R_pred_^2^), the adjusted coefficient of determination for calibration (R_pred_^2^_adj_), the root mean square error for prediction (RMSEP), the standard error of prediction (SEP), the ratio of prediction to deviation (RPD) and the ratio of the error range (RER) (Fearn, 2002).

## 3 Results and discussion

Stabilized enzymes are essential for industrial biocatalysis due to their enhanced operational stability, resulting in prolonged enzyme activity and overall cost-efficiency. In that sense, ensuring enzyme stability is a significant challenge in integrating enzymes into continuous systems and large-scale biocatalysis, as well as in minimizing enzyme inactivation during storage, reagent preparation and biocatalytic assays. Previous research groups have demonstrated DES capability to stabilize various industrially relevant dehydrogenases (Gotor-Fernández and Paul, 2019; Gajardo-Parra et al., 2023). Additionally, we recently showcased significant stabilization of the nicotinamide coenzyme utilized by these enzymes within DES (Radović et al., 2022). Given the demonstrated influence of DES composition, including starting components and water content, on the stability of dehydrogenases, we were compelled to broaden the scope of DES experimental screening for a more nuanced understanding of DES stabilization capability. For that purpose, a total of 28 DES were prepared and the σ-profile of each DES was calculated to develop a straightforward and reliable QSPR-ANN model for predicting the stability of these three enzymes in DES. This endeavor aimed to highlight the potential of a combined experimental and theoretical approach in guiding the rational design of DES and underscore its significance in studies concerning enzyme stability within these solvents. We assessed the capability of a diverse array of DES to stabilize NADH-dependent alcohol dehydrogenases (ADHs), specifically ADH-A from *R. ruber* and Lk-ADH from *L. kefir*, widely utilized for stereoselective ketone reduction (Bradshaw et al., 1992; Pȩkala en Zelaszczyk, 2009; Karabec et al., 2010), along with glucose dehydrogenase GDH from *Bacillus megaterium*, a versatile tool for nicotinamide adenine dinucleotide (NAD(P)H) regeneration in enzyme-catalyzed ketone reduction.

### 3.1 DES selection and σ-descriptor definition

For clarity in data interpretation, DES were roughly divided into two categories according to the chosen HBA, namely, betaine-based and choline chloride-based DES (Table 1). The choice of HBD such as ethylene glycol, glycerol and propylene glycol was influenced by promising studies recognizing polyol-based DES as effective stabilizers for dehydrogenases and enzymes in general (Bittner et al., 2022; Gajardo-Parra et al., 2023). Consequently, DES containing urea or both urea and polyol were also added to assess their individual or combined effect on enzymatic stability. The inclusion of urea in polyol-based DES builds upon our prior findings, which suggested that urea, acting as a HBD, significantly enhances the stabilization of the NAD coenzyme (Radović et al., 2022). Finally, various water contents were investigated for each DES, specifically 10, 30 and 50 wt%. DES dilutions beyond 50% were not considered, as additional water would result in a solvent behaving more like a solution of its individual components in water (Gutiérrez et al., 2021).

For the purpose of developing QSPR models (Section 3.3), the σ-profile of each DES was calculated by using BOVIA COSMOtherm software. The σ-profile shows the probability of finding surface segments with specific σ polarities on a molecule’s surface. It contains the essential chemical information to predict a compound’s electrostatic interactions, hydrogen bonding and dispersion forces. The charge distribution and the width and height of peaks in the σ-profile vary with the nature of the molecules, allowing for quantitative assessment of any structural changes, making them extremely useful in deep learning to accurately correlate and predict a wide range of physicochemical properties (Abranches et al., 2022; Panić et al., 2022). Herein, the σ-profile curves for each HBA and HBD were divided into 10 regions and the area under each region was calculated, taking into account the molar ratios of the components and the water content (Supplementary Table S1). As evident from Figure 1, the variation in HBD and water proportions led to the formation of DES with distinct molecular polarity distributions. This property can be quantitatively represented by the DES σ-profile - a mixture’s polar surface charge on the polarity scale (calculated by BOVIA COSMOtherm software) (Klamt, 1995). Typically, HBA exhibits peaks in the negative potential region, HBD peaks in the positive potential region and nonpolar molecules peaks in the potential region around zero (Figure 1B). Figure 1C clearly shows that even small changes in DES composition, such as increasing the water content from 10 to 50 wt%, drastically influence the overall DES σ-profile. This illustrates the capability of the molecular descriptors generated by the software to capture nuanced phenomena such as polarizability and asymmetry in electron density. These characteristics are pivotal for comprehensively exploring and quantifying the expansive chemical landscape of these solvents, which all potentially have a detrimental effect on enzyme behavior.

### 3.2 Experimental assessment of DES influence on enzyme stability

**FIGURE 2:**
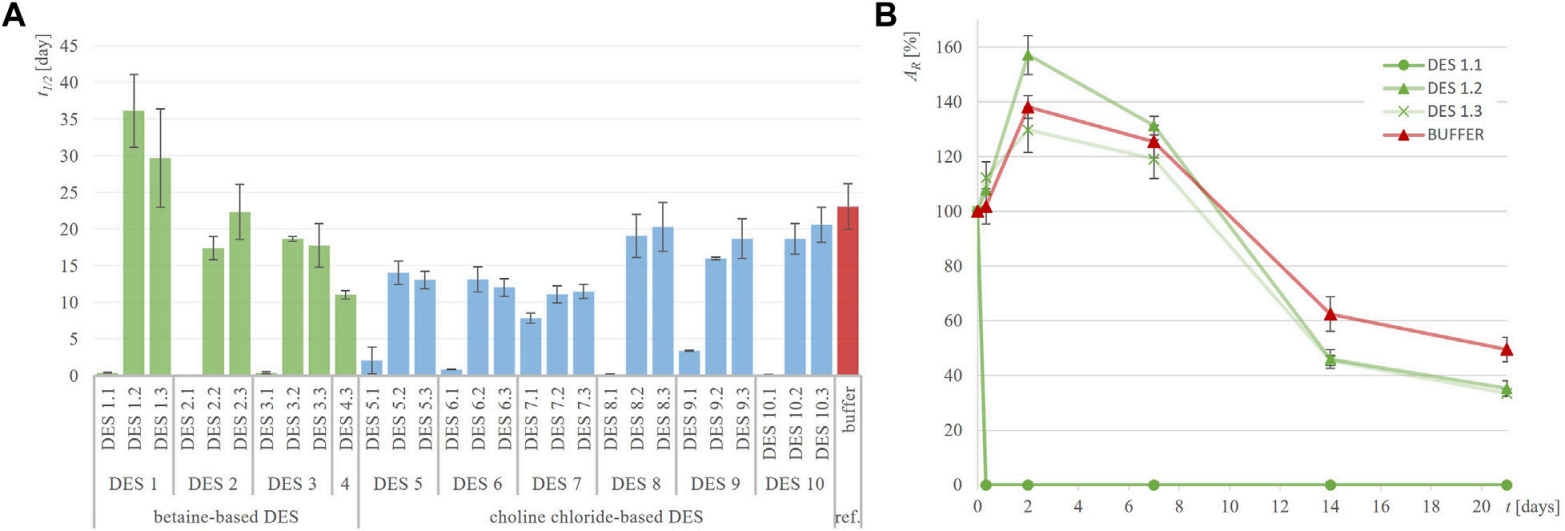
Half-life of ADH-A (*t*_*1/2*_, days) in different DES **(A)** and exemplary residual ADH-A activity (*A*_*R*_, %) over time in DES 1 **(B)** compared to 50 mM TRIS-HCl buffer, pH = 7.5.

**FIGURE 3:**
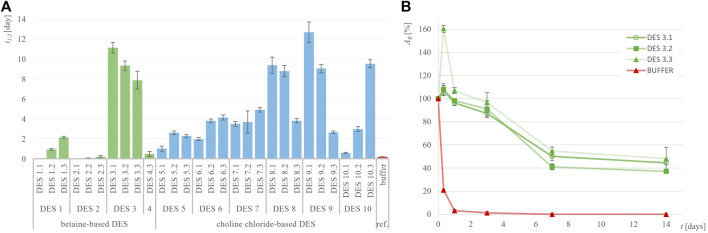
Half-life of GDH (*t*_*1/2*_, days) in different DES **(A)** and exemplary residual GDH activity (*A*_*R*_, %) over time in DES 3 **(B)** compared to 50 mM potassium-phosphate buffer, pH = 7.5.

**FIGURE 4:**
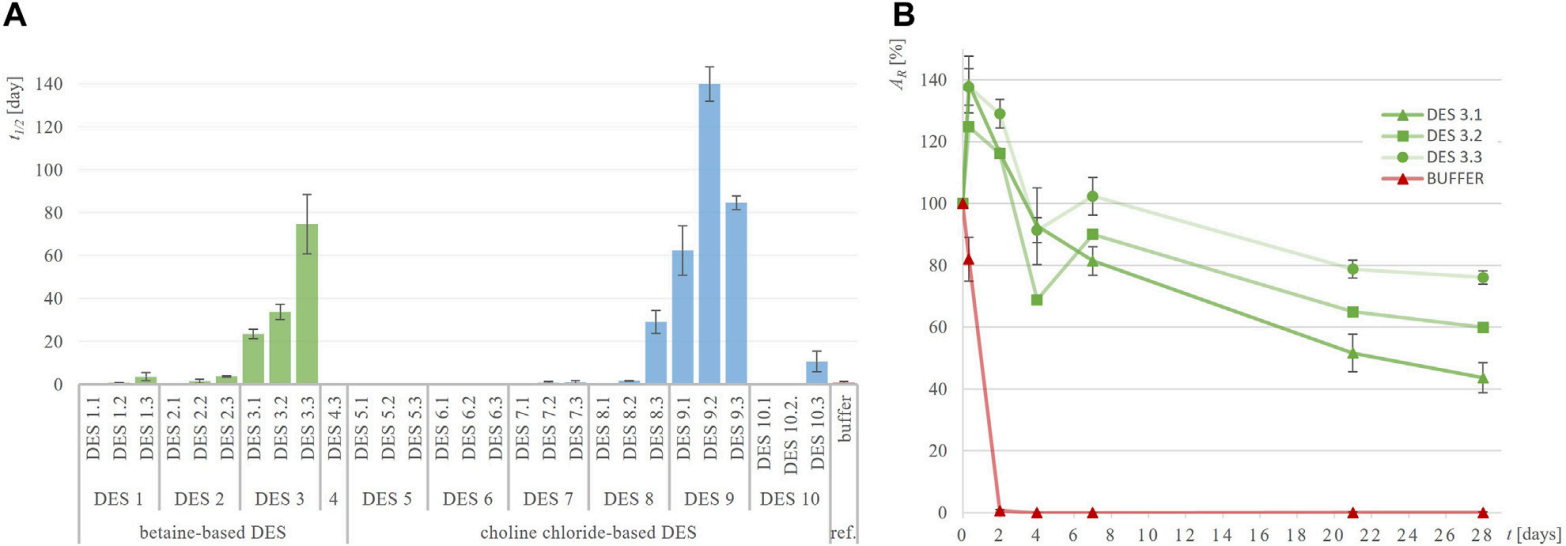
Half-life of Lk-ADH (*t*_*1/2*_, days) in different DES **(A)** and exemplary residual Lk-ADH activity (*A*_*R*_, %) over time in DES 3 **(B)** compared to 50 mM TRIS-HCl buffer, pH = 7.5.

To determine whether DES are effective in suppressing the inactivation of the three selected dehydrogenases, the residual enzymatic activity during incubation in 28 different DES and their corresponding reference buffers at 30°C was measured utilizing model activity assay (Section 2.4). Considering that the selected dehydrogenases have the potential to oxidize hydroxyl groups present in some HBDs (ethylene glycol, glycerol and propylene glycol), we investigated whether the enzymes not only oxidize the substrates used in the assays but also the DES components. By substituting the model substrates used in the assays with pure DES components, we examined whether NADH formation occurred. No turnover of cofactors was detected, confirming that the model activity assays can be applied to the entire experimental setup (data not shown). While investigating the stability of dehydrogenases in their optimal reference buffer, it is evident that they do not share the same stabilization issues: a comparison of their individual stabilities in the reference buffer reveals notable differences (Table 2). Specifically, ADH-A demonstrates high stability in the reference buffer, retaining up to 50% of residual activity even after 21 days of incubation. On the other hand, Lk-ADH and GDH are good candidates for prolonged stability enhancements. Unlike ADH-A, they experience a complete loss of activity in the reference buffer by the fourth and seventh day of incubation. Replacing reference buffers with DES resulted in varying trends in residual enzymatic activities (Supplementary Figures S1-S3), depending on the enzyme. For all enzymes, both in reference buffers and DES, inactivation followed first-order kinetics. Therefore, such kinetic model was used to calculate enzymes’ inactivation rate constants (*k,* h^-1^) (Supplementary Table S2) and the corresponding enzymes’ half-lifes (*t*_1/2_, day) (Figures 2–4). It is important to note that in most cases, the residual enzymatic activity increased from initial value of 100% within the first few days of incubation in DES, followed by a gradual decrease. This phenomenon of enzyme “overstabilization” in DES has been previously reported for laccase and hydrolytic enzymes (Delorme et al., 2020; Varriale et al., 2022; Guo et al., 2023). The authors of these studies explained it as the enzymes needing time to uniformly distribute through the viscous medium or to undergo certain rearrangements of their secondary and tertiary structures while adapting to the DES environment. However, as none of these explanations were applicable to the setup used in this study (incubated enzyme’s activity measured in buffer), for the sake of developing reliable kinetic models, values for residual activities exceeding 100% were omitted. Finally, for clarity, the results for each enzyme’s behavior are first analyzed individually. This is followed by a comprehensive discussion, with a special emphasis on the relationship between DES structure and enzyme stability.

**TABLE 2:** Residual activity (*A*_*R*_, %) and half-life (*t*_*1/2*_, day) of dehydrogenases in reference buffers.

enzyme	Time (days)									*t*_1/2_ ± st. dev. (day)
	0	0.1	1	2	3	4	7	14	21	
ADH-A^a^	100	112	120	138	138	133	126	62	50	23.038 ± 3.122
GDH^b^	100	92	3	2	1	1	0	0	0	0.193 ± 0.009
Lk-ADH^a^	100	78	6	1	1	0	0	0	0	0.796 ± 0.546

^a^ 50 mM TRIS-HCl, pH = 7.5.

^b^ 50 mM potassium-phosphate buffer, pH = 7.5.

Figure 2; Supplementary Figure S1 illustrate the notable stability of ADH-A in various DES and the reference buffer, persisting for up to 21 days. This observation is consistent with previous findings indicating that this enzyme displays high stability in non-conventional media, particularly organic solvents (Karabec et al., 2010). Such stability comes in handy when biocatalysis of hydrophobic substrates is performed that often requires substantial amounts of organic solvents typically inadequate for enzymes. Predicting the industrial demand for a fast adaptation to growing environmental challenges, DES are hereon being explored as novel green solvents intended to replace currently used organic solvents. As shown in Figure 2, incubating ADH-A in different DES during the period of 21 days at 30°C revealed that, in general, most of the DES containing 30 and 50 wt% water provided equal or slightly lower stabilization for ADH-A. Residual ADH-A activity after the incubation period ranged from 20% to 50% (*t*_1/2_ from 11.0 to 36.1 days) compared to the reference buffer, where residual ADH-A activity was 50%, with *t*_1/2_ = 23 days. Only in two betaine-based DES with ethylene glycol as HBD, namely, DES 1.2 and DES 1.3, an improvement in the enzyme stability compared to the reference buffer was observed, with *t*_1/2_ values of 36.1 and 29.7 days, respectively. It is worth noting that after the same incubation period, ADH-A activity was completely lost in all DES containing 10 wt% water with *t*_1/2_ < 7.8 days.

GDH has been identified as an enzyme requiring significant stabilization improvement, as its residual activity drastically dropped by 97% after just 1 day of incubation in the reference buffer and was completely lost after only 4 days at 30°C, giving a *t*_1/2_ of 0.2 days (Figure 3). However, incubating GDH in various DES over the same period revealed that nearly all tested DES provided significantly greater stabilization than the reference buffer. This effect was most pronounced in polyol-based DES, with DES 3.1 showing 45%, DES 3.3 showing 48%, DES 8.1 showing 47% and DES 10.3 showing 46% residual activity after 14 days of incubation (Supplementary Figure S2). Comparing the half-life of GDH in the reference buffer (*t*_1/2_ = 0.2 days) with those in DES 3.1 (*t*_1/2_ = 11.1 days) and DES 9.1 (*t*_1/2_ = 12.7 days), an astonishing 60-fold approximate increase was observed. Interestingly, in contrast to ADH-A, in DES 3, DES eight and DES 9, this enzyme was particularly stabilized at low water content of 10 wt%.

Lk-ADH is an (*R*)-specific alcohol dehydrogenase that exhibits significant stability issues in the reference buffer at 30°C. As shown in Figure 4; Supplementary Figure S3, Lk-ADH lost 99% of its residual activity after just 2 days of incubation in buffer. Efforts to enhance this poor stability by using DES were largely successful. Several DES significantly stabilized the enzyme, retaining more than 50% of its residual activity even after 28 days of incubation. Specifically, DES 3.2, 3.3, and 9.1 maintained residual activities of 60%, 76% and 56%, respectively. Notably, the half-life of Lk-ADH in the reference buffer (*t*_1/2_ = 0.8 days) increased dramatically in DES 3.2 (*t*_1/2_ = 33.7 days) and DES 3.3 (*t*_1/2_ = 74.6 days), representing up to a 90-fold improvement. The addition of water to DES generally enhanced the medium’s ability to stabilize the enzyme.

Having examined the specific impacts of DES on dehydrogenase stability and considering the results presented, several overarching conclusions can be drawn and discussed. Overall, DES demonstrate significant potential in stabilizing enzymes Lk-ADH and GDH, which are inherently unstable in aqueous buffered solutions. However, for ADH-A, an enzyme that is relatively stable in such environments, stabilization improvements were observed primarily in betaine-based DES featuring ethylene glycol as the HBD (DES 1). When we analyze the molecules used as HBA or HBD, it seems that choline chloride-based DES are generally more suitable for long-term enzyme incubations than betaine-based ones. In depth, DES 3 (B:Gly), DES 8 (ChCl:EG), DES 9 (ChCh:Gly) and DES 10 (ChCh:PG), all containing exclusively polyols as HBD, emerge as top candidates for all tested enzymes. This aligns with literature findings that recognize polyol-based DES, especially glycerol-based ones, as excellent stabilizers for dehydrogenases and other enzymes relevant to industry (Bittner et al., 2022; Gajardo-Parra et al., 2023). Specifically, observed beneficial effect is attributed to the abundance of hydroxyl (OH) groups in the HBD: the greater the number of these groups, the better, as they enhance enzyme stability by forming hydrogen bonds with the amino acids of the enzyme (Toledo et al., 2019; Delorme et al., 2020). More specifically, by studying the mechanism of lysozyme stabilization in choline chloride-based DES with glycerol as a HBD using molecular dynamics, Hebbar et al. detected a high number of protein-glycerol interactions, which could be attributed to the numerous hydrogen bond-donating functional groups (Hebbar et al., 2023). This DES also induced a sharp decrease in intra-protein interactions and an increase in protein-choline hydrogen bonds, indicating a change in the overall protein conformation. Additionally, Sanchez-Fernandez et al. showed that the same DES forms a protective hydration layer around lysozyme, thus retaining its globular nature and backbone rigidity, even though changes in the secondary structure were observed (Sanchez-Fernandez et al., 2022). Regarding urea, another HBD tested in this study, the results demonstrated its limited capacity to stabilize the three tested enzymes, whether used as the sole HBD or as part of a three-component DES. Nevertheless, DES incorporating this amide were able to stabilize the enzymes as effectively as, or even better than, the corresponding reference buffer. Furthermore, the results indicate that the stability of the tested dehydrogenases is significantly influenced by the water content in DES. Generally, all three enzymes exhibit faster activity loss in DES with the lowest water content (10 wt%), while in DES containing 30 or 50 wt% of water, the enzyme inactivation rates were similar. This suggests that the selected enzymes generally require more than 10% water in DES to maintain their structural integrity. These findings align with previous observations that most oxidoreductases, including the selected alcohol dehydrogenases, require small amounts of water in DES to retain their activity (Mourelle-Insua et al., 2019; Bittner et al., 2022). Namely, it is well established that the water content in the enzyme’s environment directly influences its hydration level and, consequently, its structural integrity, crucial for maintaining enzymes in an active and stable state. In this context, DES are unique solvents: at low water content, they effectively absorb water into their hydrogen bond-based supramolecular network. For example, small amounts of water in hydrated DES can enhance the hydrogen bond network by integrating water molecules into the DES voids, acting as a small HBD or HBA. This phenomenon has been confirmed by López-Salas et al., who demonstrated that in a “water-in-DES” system with up to about 40 wt% water, the tetrahedral structure of water is distorted due to its incorporation into the hydrogen bond complexes formed among the original DES components (López-Salas et al., 2019). Consequently, this phenomenon reduces the availability and activity of water molecules in the medium (DES), potentially leading to enzyme dehydration to a level where irreversible denaturation occurs (as herein evidenced by a complete loss of activity upon returning the enzymes to buffered aqueous medium).

Lastly, if we consider DES as a medium for a long-term storage of the enzymes, a significant advantage of these solvents is their resistance to contamination. Typically, incubation of enzymes in aqueous solutions at 30°C is susceptible to contamination, which was observed in this research as well. Enzyme samples prepared in buffers exhibited turbidity and sensory changes. Conversely, no contamination was detected in the DES samples, further underscoring the robustness of these solvents for long-term enzyme storage. This finding highlights the stability and integrity of DES, making them an ideal medium for maintaining enzyme activity over extended periods without the risk of inactivation or contamination.

### 3.3 Development of QSPR-ANN models using DES molecular descriptors

Our next step was to model the relationship between the enzyme inactivation rate constant and DES descriptors (QSPR model) for each enzyme based on the experimental data, utilizing ANNs. First, the first-order kinetic model was used to compare the inactivation rate constants over time for all three analyzed enzymes (Supplementary Table S2). By analyzing the enzymes’ inactivation rate constants, we can quantify the influence of DES on enzyme activity. For ADH-A, the highest inactivation rate constant was observed with DES 2.1 (*k* = 0.9639 h⁻^1^), while the lowest constant was obtained with DES 1.2 (*k* = 0.0008 h⁻^1^). This difference is directly linked to DES composition: although both DES contain the same HBA (betaine) and similar HBD (polyols - ethylene glycol and propylene glycol), DES 2.1 contains only 10 wt% water while DES 1.2 contains 30% water. For GDH, the lowest inactivation rate constant was found with DES 9.1 (*k* = 0.0023 h⁻^1^), while for Lk-ADH, the lowest rate constant was with DES 9.2 (*k* = 0.0002 h⁻^1^). However, some DES resulted in very high inactivation rate constants for GDH and Lk-ADH (up to 50.9799 and 51.0000 h⁻^1^, respectively). For GDH, this was observed with DES 1.1 and DES 2.1, while for Lk-ADH, high inactivation rates were noted across multiple DES, specifically DES 1.1, DES 6.2, DES 6.3, DES 8.1, DES 10.1 and DES 10.2.

The number of input variables was selected based on the correlation matrix between inactivation rate constants and 10 DES descriptors. Table 3 shows the ANNs selected as optimal for predicting specific inactivation rate constants based on R^2^ and RMSE for the training, test and validation datasets, as well as considering the number of neurons in the hidden layer. A lower number of neurons in the hidden layer was considered advantageous as it implies a simpler network structure (Chen et al., 2022). Developed models were used for independent prediction to analyze their applicability. Results showed that the best agreement between the experimental data and the data predicted by the ANN model was obtained for ADH-A (R_pred_^2^ = 0.99904, R_pred_^2^_adj_ = 0.9902, RMSEP = 0.0016, SEP = 0.0004, RPD = 3.0466, RER = 7.9065), followed by GDH (R_pred_^2^ = 0.9021, R_pred_^2^_adj_ = 0.8655, RMSEP = 0.0051, SEP = 0.0011, RPD = 2.4902, RER = 7.7491) and Lk-ADH (R_pred_^2^ = 0.8927, R_pred_^2^_adj_ = 0.8524, RMSEP = 0.0066, SEP = 0.0032, RPD = 1.1976, RER = 4.1260). Based on R_pred_^2^, all developed ANN models can be considered substantial (R_pred_^2^ > 0.75) (Hussain et al., 2018). The suitability of the developed ANN models for predicting the inactivation rate constants based on DES descriptors was also estimated using the ratio of prediction to deviation (RPD) and the ratio of the error range (RER). Models with RPD<1.4 are considered non-reliable, those with RPD in the range from 1.4 to two are considered fair, while models with RPD>2 are described as excellent models (Chang et al., 2001). Furthermore, models with RER>4 are acceptable for data screening, models with RER>10 can be used for quality control, while models with RER >15 can be used for quantification (Sim et al., 2023). Based on the obtained results, it can be concluded that developed ANN models for ADH-A and GDH can be considered excellent (RPD>2) and can be used for screening (4<RER<11) the inactivation rate constants based on DES descriptors. To make the model useable for quality control, the DES descriptor database should sourly expand to more DES with different HBA and HBD. As mentioned by Lemaoui et al. a significant advantage of DESs is their tailor-made nature, leading to an extensive array of potential DES variants (Lemaoui et al., 2022). Hence, computational methods capable of predicting the properties of DESs are crucial for numerous industrial applications and research endeavors.

**TABLE 3:** Neural network calibration and prediction parameters.

		Calibration					Prediction					
Enzyme	Network	Training perf./error	Test perf./error	Validation perf./error	Hidden activation	Output activation	R_pred_^2^	R_pred_^2^_adj_	RMSEP	SEP	RPD	RER
ADH-A	MLP 8-6-1	0.9994/0.0001	0.9965/0.0002	0.9919/0.0007	Exponential	Tanh	0.9904	0.9902	0.0016	0.0004	3.047	7.907
GDH	MLP 6-5-1	0.9984/0.0002	0.9645/0.0002	0.9423/0.0003	Identity	Logistic	0.9021	0.8655	0.0051	0.0011	2.490	7.749
Lk-ADH	MLP 3-6-1	0.9907/0.0007	0.9428/0.0008	0.9082/0.0008	Identity	Logistic	0.8927	0.8524	0.0066	0.0032	1.198	4.126

Abbreviations: multilayer perceptron (MPL), root mean square error for prediction (RMSEP), standard error of prediction (SEP), ratio of prediction to deviation (RPD) and ratio of the error range (RER).

## 4 Conclusion

This study demonstrates the significant potential of DES for stabilizing tested dehydrogenases, highlighting their capacity to enhance enzyme stability and operational efficiency in industrial biocatalysis. By systematically screening 28 different DES and developing robust ANN models, we identified DES based on polyols as particularly effective stabilizers, significantly outperforming reference buffers. Furthermore, we demonstrated that the ANN models, with their high predictive accuracy, provide a dependable means for *in silico* screening of DES, bypassing the need for labour-intensive experimental screening and paving the way for the rational design of tailored DES formulations. Also, such models enable the exploration of a vast chemical space of DES that would be impractical to cover experimentally. In conclusion, the presented integrated experimental and *in silico* approach offers a way to harness the strengths of both methods, thereby enhancing reliability, optimizing resources, and accelerating the development of DES for enzyme stabilization.

## Data Availability

The original contributions presented in the study are included in the article/Supplementary Material, further inquiries can be directed to the corresponding authors.

## References

[B1] Abranches D. O. Coutinho J. A. P. (2023). Everything you wanted to know about deep eutectic solvents but were afraid to Be told. Annu. Rev. Chem. Biomol. Eng. 14, 141–163. 10.1146/annurev-chembioeng-101121-085323 36888992

[B2] Abranches D. O. Larriba M. Silva L. P. Melle-Franco M. Palomar J. F. Pinho S. P. (2019). Using COSMO-RS to design choline chloride pharmaceutical eutectic solvents. Fluid Phase Equilib. 497, 71–78. 10.1016/j.fluid.2019.06.005

[B3] Abranches D. O. Zhang Y. Maginn E. J. Colón Y. J. (2022). Sigma profiles in deep learning: towards a universal molecular descriptor. Chem. Commun. 58, 5630–5633. 10.1039/D2CC01549H 35438096

[B4] Asghar S. Z. Kaviani R. Shayanfar A. (2023). Solubility of some drugs in aqueous solutions of choline chloride-based deep eutectic solvent systems: experimental data, modeling, and the impact of solution pH. Iran. J. Pharm. Res. IJPR 22, 137011. 10.5812/IJPR-137011 PMC10728849 38116558

[B5] Benguerba Y. Alnashef I. M. Erto A. Balsamo M. Ernst B. (2019). A quantitative prediction of the viscosity of amine based DESs using Sσ-profile molecular descriptors. J. Mol. Struct. 1184, 357–363. 10.1016/j.molstruc.2019.02.052

[B6] Bittner J. P. Zhang N. Huang L. Domínguez De María P. Jakobtorweihen S. Kara S. (2022). Impact of deep eutectic solvents (DESs) and individual DES components on alcohol dehydrogenase catalysis: connecting experimental data and molecular dynamics simulations. Green Chem. 24, 1120–1131. 10.1039/d1gc04059f

[B7] Bradshaw C. W. Wong C. H. Hummel W. (1992). Lactobacillus kefir alcohol dehydrogenase: a useful catalyst for synthesis. J. Org. Chem. 57, 1532–1536. 10.1021/jo00031a037

[B8] Chang C.-W. Laird D. A. Mausbach M. J. Hurburgh C. R. (2001). Near‐Infrared reflectance spectroscopy–principal components regression analyses of soil properties. Soil Sci. Soc. Am. J. 65, 480–490. 10.2136/SSSAJ2001.652480X

[B9] Chen X. Zheng H. Wang H. Yan T. (2022). Can machine learning algorithms perform better than multiple linear regression in predicting nitrogen excretion from lactating dairy cows. Sci. Rep. 12, 12478–12513. 10.1038/s41598-022-16490-y 35864287 PMC9304409

[B10] Delorme A. E. Andanson J. M. Verney V. (2020). Improving laccase thermostability with aqueous natural deep eutectic solvents. Int. J. Biol. Macromol. 163, 919–926. 10.1016/j.ijbiomac.2020.07.022 32650014

[B11] Fearn T. (2002). Assessing calibrations: SEP, RPD, RER and R2. NIR news 13, 12–13. 10.1255/NIRN.689

[B12] Gajardo-Parra N. F. Rodríguez G. Arroyo-Avirama A. F. Veliju A. Happe T. Canales R. I. (2023). Impact of deep eutectic solvents on kinetics and folding stability of formate dehydrogenase. Processes 11, 2815. 10.3390/pr11102815

[B13] García G. Aparicio S. Ullah R. Atilhan M. (2015). Deep eutectic solvents: physicochemical properties and gas separation applications. Energy Fuels 29, 2616–2644. 10.1021/ef5028873

[B14] Gotor-Fernández V. Paul C. E. (2019). Deep eutectic solvents for redox biocatalysis. J. Biotechnol. 293, 24–35. 10.1016/j.jbiotec.2018.12.018 30690099

[B15] Guo J. Zhao N. Zhao Y. Jin H. Sun G. Yu J. (2023). Effect of deep eutectic solvents on the activity and stability of cellulases and pectinases. ACS Omega 8, 45678–45686. 10.1021/acsomega.3c06088 38075793 PMC10701881

[B16] Gutiérrez A. Atilhan M. Aparicio S. (2021). Molecular dynamics study on water confinement in deep eutectic solvents. J. Mol. Liq. 339, 116758. 10.1016/J.MOLLIQ.2021.116758

[B17] Hebbar A. Dey P. Vatti A. K. (2023). Lysozyme stability in various deep eutectic solvents using molecular dynamics simulations. J. Biomol. Struct. Dyn., 1–9. 10.1080/07391102.2023.2275178 37909488

[B18] Huang L. Bittner J. P. Domínguez de María P. Jakobtorweihen S. Kara S. (2020). Modeling alcohol dehydrogenase catalysis in deep eutectic solvent/water mixtures. ChemBioChem 21, 811–817. 10.1002/CBIC.201900624 31605652 PMC7154551

[B19] Hussain S. Fangwei Z. Siddiqi A. F. Ali Z. Shabbir M. S. (2018). Structural equation model for evaluating factors affecting quality of social infrastructure projects. Sustainability 10, 1415. 10.3390/SU10051415

[B20] Juneidi I. Hayyan M. Hashim M. A. Hayyan A. (2017). Pure and aqueous deep eutectic solvents for a lipase-catalysed hydrolysis reaction. Biochem. Eng. J. 117, 129–138. 10.1016/j.bej.2016.10.003

[B21] Karabec M. Łyskowski A. Tauber K. C. Steinkellner G. Kroutil W. Grogan G. (2010). Structural insights into substrate specificity and solvent tolerance in alcohol dehydrogenase ADH-‘A’ from Rhodococcus ruber DSM 44541. Chem. Commun. 46, 6314–6316. 10.1039/C0CC00929F 20676439

[B22] Klamt A. (1995). Conductor-like screening model for real solvents: a new approach to the quantitative calculation of solvation phenomena. J. Phys. Chem. 99, 2224–2235. 10.1021/j100007a062

[B23] Klamt A. Jonas V. Bürger T. Lohrenz J. C. W. (1998a). Refinement and parametrization of COSMO-RS. J. Phys. Chem. A 102, 5074–5085. 10.1021/jp980017s

[B24] Klamt A. Jonas V. Bürger T. Lohrenz J. C. W. (1998b). Refinement and parametrization of COSMO-RS. J. Phys. Chem. A 102, 5074–5085. 10.1021/jp980017s

[B25] Lampel K. A. Uratani B. Chaudhry G. R. Ramaley R. F. Rudikoff S. (1986). Characterization of the developmentally regulated Bacillus subtilis glucose dehydrogenase gene. J. Bacteriol. 166, 238–243. 10.1128/JB.166.1.238-243.1986 3082854 PMC214582

[B26] Lemaoui T. Boublia A. Darwish A. S. Alam M. Park S. Jeon B. H. (2022). Predicting the surface tension of deep eutectic solvents using artificial neural networks. ACS Omega 7, 32194–32207. 10.1021/acsomega.2c03458 36120015 PMC9475633

[B27] Lemaoui T. Darwish A. S. Hammoudi N. E. H. Abu Hatab F. Attoui A. Alnashef I. M. (2020a). Prediction of electrical conductivity of deep eutectic solvents using COSMO-RS Sigma profiles as molecular descriptors: a quantitative structure-property relationship study. Ind. Eng. Chem. Res. 59, 13343–13354. 10.1021/acs.iecr.0c02542

[B28] Lemaoui T. Hammoudi N. E. H. Alnashef I. M. Balsamo M. Erto A. Ernst B. (2020b). Quantitative structure properties relationship for deep eutectic solvents using Sσ-profile as molecular descriptors. J. Mol. Liq. 309, 113165. 10.1016/J.MOLLIQ.2020.113165

[B29] López-Salas N. Vicent-Luna J. M. Imberti S. Posada E. Roldán M. J. Anta J. A. (2019). Looking at the “water-in-deep-eutectic-solvent” system: a dilution range for high performance eutectics. ACS Sustain. Chem. Eng. 7, 17565–17573. 10.1021/acssuschemeng.9b05096

[B30] Mourelle-Insua Á. Lavandera I. Gotor-Fernández V. (2019). A designer natural deep eutectic solvent to recycle the cofactor in alcohol dehydrogenase-catalysed processes. Green Chem. 21, 2946–2951. 10.1039/c9gc00318e

[B31] Panić M. Cvjetko Bubalo M. Radojčić Redovniković I. (2021). Designing a biocatalytic process involving deep eutectic solvents. J. Chem. Technol. Biotechnol. 96, 14–30. 10.1002/jctb.6545

[B32] Panić M. Radović M. Cvjetko Bubalo M. Radošević K. Rogošić M. Coutinho J. A. P. (2022). Prediction of pH value of aqueous acidic and basic deep eutectic solvent using COSMO-RS σ profiles’ molecular descriptors. Molecules 27, 4489. 10.3390/molecules27144489 35889358 PMC9324476

[B33] Pätzold M. Siebenhaller S. Kara S. Liese A. Syldatk C. Holtmann D. (2019). Deep eutectic solvents as efficient solvents in biocatalysis. Trends Biotechnol. 37, 943–959. 10.1016/J.TIBTECH.2019.03.007 31000203

[B34] Pekala E. Zelaszczyk D. (2009). Alcohol dehydrogenases as tools for the preparation of enantiopure metabolites of drugs with methyl alkyl ketone moiety. Sci. Pharm. 77, 9–17. 10.3797/SCIPHARM.0901-26

[B35] Radović M. Hok L. Panić M. Cvjetko Bubalo M. Vianello R. Vinković M. (2022). Deep eutectic solvents as a stabilising medium for NAD coenzyme: unravelling the mechanism behind coenzyme stabilisation effect. Green Chem. 24, 7661–7674. 10.1039/d2gc02656b

[B36] Sanchez-Fernandez A. Basic M. Xiang J. Prevost S. Jackson A. J. Dicko C. (2022). Hydration in deep eutectic solvents induces non-monotonic changes in the conformation and stability of proteins. J. Am. Chem. Soc. 144, 23657–23667. 10.1021/jacs.2c11190 36524921 PMC9801427

[B37] Sim J. McGoverin C. Oey I. Frew R. Kebede B. (2023). Near-infrared reflectance spectroscopy accurately predicted isotope and elemental compositions for origin traceability of coffee. Food Chem. 427, 136695. 10.1016/J.FOODCHEM.2023.136695 37385064

[B38] Toledo M. L. Pereira M. M. Freire M. G. Silva J. P. A. Coutinho J. A. P. Tavares A. P. M. (2019). Laccase activation in deep eutectic solvents. ACS Sustain. Chem. Eng. 7, 11806–11814. 10.1021/acssuschemeng.9b02179

[B39] Varriale S. Delorme A. E. Andanson J. M. Devemy J. Malfreyt P. Verney V. (2022). Enhancing the thermostability of engineered laccases in aqueous betaine-based natural deep eutectic solvents. ACS Sustain. Chem. Eng. 10, 572–581. 10.1021/acssuschemeng.1c07104 35036179 PMC8753991

[B40] Wang J. Song Z. Chen L. Xu T. Deng L. Qi Z. (2021). Prediction of CO2 solubility in deep eutectic solvents using random forest model based on COSMO-RS-derived descriptors. Green Chem. Eng. 2, 431–440. 10.1016/J.GCE.2021.08.002

[B41] Zhang N. Domínguez de María P. Kara S. (2024). Biocatalysis for the synthesis of active pharmaceutical ingredients in deep eutectic solvents: state-of-the-art and prospects. Catal 14, 84–14. 10.3390/CATAL14010084

[B42] Zhang Q. De Oliveira Vigier K. Royer S. Jérôme F. (2012). Deep eutectic solvents: syntheses, properties and applications. Chem. Soc. Rev. 41, 7108–7146. 10.1039/C2CS35178A 22806597

