# Supplementary material for: Rational design of deep eutectic solvents for the stabilization of dehydrogenases: an artificial neural network prediction approach
Source: Front Chem. 2024 Aug 1;12:1436049. doi: 10.3389/fchem.2024.1436049 (PMC11325221; doi:10.3389/fchem.2024.1436049)
Supplement: Supplementary file 1 [file DataSheet1.PDF]

Supplementary Material

Supplementary Material – Enzyme production

Glucose dehydrogenase *Bacillus megaterium* (GDH, internal plasmid number pEG521) was expressed in *E. coli* BLE21(DE3) in a pET28a(+) vector with a His6 at the N-terminus.

AA-sequence:
HMMYPDLKGKVVAITGAASGLGKAMAIRFGKEQAKVVINYYSNKQDPNEVKEEVIKAGGE
AVVVQGDVTKEEDVKNIVQTAIKEFGTLDIMINNAGLENPVPSHEMPLKDWDKVIGTNLTG
AFLGSREAIKYFVENDIKGNVINMSSVHAFPWPLFVHYAASKGGIKLMTETLALEYAPKGIR
VNNIGPGAINTPINAEKFADPKQKADVESMIPMGYIGEPEEIAAVAAWLASKEASYVTGITLF
ADGGMTQYPSFQAGRG

DNA-sequence:

catATGATGTACCCCGATCTTAAAGGCAAAGTTGTGGCGATCACCGGAGCGGCGTCCGGA
TTGGGTAAAGCCATGGCGATCCGTTTTGGAAAAGAACAAGCGAAGGTCGTGATTAATTA
CTACTCGAACAAGCAGGACCCTAATGAAGTTAAAGAGGAAGTTATTAAAGCAGGTGGT
GAGGCCGTCGTAGTGCAGGGTGACGTGACTAAGGAAGAGGACGTTAAGAACATTGTTC
AGACTGCGATCAAAGAATTTGGGACTCTGGACATCATGATCAACAACGCAGGCTTAGAG
AACCCGGTTCCGTCGCATGAGATGCCTTTGAAAGACTGGGACAAGGTAATTGGTACTAA
TCTGACCGGCGCATTCCTGGGCTCTCGCGAGGCAATCAAGTACTTTGTGGAGAATGACA
TTAAAGGCAACGTGATCAATATGTCTTCAGTTCATGCATTCCCCTGGCCCCTGTTCGTGC
ATTACGCTGCCAGCAAGGGTGGCATTAAACTTATGACCGAGACCTTGGCCTTAGAGTAT
GCCCCTAAAGGGATCCGTGTGAACAACATCGGCCCTGGCGCTATTAATACCCCGATTAA
CGCAGAGAAGTTTGCCGATCCGAAGCAAAAGGCGGACGTGGAGAGTATGATCCCGATG
GGCTACATTGGAGAGCCCGAAGAAATTGCGGCGGTTGCCGCATGGTTAGCAAGCAAAG
AGGCAAGTTATGTGACGGGTATTACCCTCTTTGCCGATGGTGGAATGACTCAGTACCCGT
CGTTTCAAGCCGGTCGTGGCTGActcgag

Alcohol dehydrogenase from *Lactobacillus kefir* (Lk-ADH, plasmid number pEG326) was expressed in *E. coli* BLE21(DE3)pLysS in a pET21 vector.

AA-sequence:

MTDRLKGKVAIVTGGTLGIGLAIADKFVEEGAKVVITGRHADVGEKAAKSIGGTDVIRFVQH
DASDEAGWTKLFDTTEEAFGPVTTVVNNAGIAVSKSVEDTTTEEWRKLLSVNLDGVFFGTR
LGIQRMKNKGLGASIINMSSIEGFVGDPTLGAYNASKGAVRIMSKSAALDCALKDYDVRVN
TVHPGYIKTPLVDDLEGAEEMMSQRTKTPMGHIGEPNDIAWICVYLASDESKFATGAEFVV
DGGYTAQ

DNA-sequence:

CATATGACCGATCGTCTGAAAGGTAAAGTTGCAATTGTTACCGGTGGCACCCTGGGTAT
TGGTCTGGCAATTGCAGATAAATTTGTTGAAGAAGGTGCCAAAGTTGTTATTACCGGTC
GTCATGCAGATGTTGGTGAAAAAGCAGCAAAAAGCATTGGTGGCACCGATGTTATTCGT
TTTGTTCAGCATGATGCAAGTGATGAAGCAGGTTGGACCAAACTGTTTGATACCACCGA
AGAAGCATTTGGTCCGGTTACCACCGTTGTTAATAATGCAGGTATTGCAGTTAGCAAAA
GCGTTGAAGATACCACCACAGAAGAATGGCGTAAACTGCTGAGCGTTAATCTGGATGGT
GTTTTTTTTGGCACCCGTCTGGGTATTCAGCGTATGAAAAACAAAGGTCTGGGTGCCAGC
ATTATCAATATGAGCAGCATTGAAGGTTTTGTTGGTGATCCGACCCTGGGTGCATATAAT
GCAAGCAAAGGTGCAGTTCGTATTATGAGCAAAAGTGCAGCACTGGATTGTGCCCTGAA
AGATTATGATGTTCGTGTTAATACCGTTCACCCTGGCTATATCAAAACACCGCTGGTTGA
TGATCTGGAAGGTGCCGAAGAAATGATGAGCCAGCGTACCAAAACCCCGATGGGTCAT
ATTGGTGAACCGAATGATATTGCCTGGATTTGTGTTTATCTGGCCAGTGATGAAAGTAAA
TTTGCGACCGGTGCCGAATTTGTTGTTGATGGTGGTTATACCGCACAGTAACTCGAG

Supplementary Table S1. List of σ-profiles, DES molecular descriptors

	Abbrev. (HBA:HBD)	Molar ratio	DES code	DES σ-profiles									
				S^1	S^2	S^3	S^4	S^5	S^6	S^7	S^8	S^9	S^{10}
1	B:EG	1:2	DES 1.1	0,00	3,80	9,37	23,37	21,37	6,41	5,62	8,16	12,01	2,02
			DES 1.2	0,00	5,04	9,69	13,71	12,03	4,69	4,86	6,73	10,19	1,32
			DES 1.3	0,00	5,65	9,85	8,95	7,42	3,84	4,48	6,03	9,30	0,97
2	B:PG	1:3	DES 2.1	0,00	4,01	8,89	19,23	27,03	11,14	6,03	8,02	11,00	1,55
			DES 2.2	0,00	5,18	9,45	11,40	14,64	7,02	5,04	6,63	9,65	1,07
			DES 2.3	0,00	5,25	8,81	5,46	3,37	2,57	3,50	4,66	7,81	0,83
3	B:Gly	1:2	DES 3.1	0,00	5,65	10,97	21,58	22,63	8,23	6,38	10,67	12,21	1,91
			DES 3.2	0,00	6,02	10,48	12,15	11,94	5,43	5,16	7,83	10,15	1,22
			DES 3.3	0,00	6,18	10,26	7,97	7,20	4,19	4,63	6,57	9,24	0,91
4	B:U	1:3	DES 4.3	0,00	6,21	11,65	7,03	4,69	4,44	4,53	6,29	9,77	0,89
5	ChCl:U	1:2	DES 5.1	0,00	5,47	15,90	19,53	11,69	7,90	6,16	10,87	15,42	0,29
			DES 5.2	0,00	5,91	12,97	11,54	6,94	5,39	5,11	8,06	11,86	0,43
			DES 5.3	0,00	6,12	11,58	7,75	4,68	4,20	4,61	6,72	10,16	0,50
6	ChCl:U:EG	1:2:2	DES 6.1	0,00	5,11	13,05	18,06	15,14	7,94	6,43	10,20	13,10	0,24
			DES 6.2	0,00	5,70	11,60	11,10	8,93	5,51	5,30	7,82	10,79	0,40
			DES 6.3	0,00	6,00	10,87	7,59	5,80	4,29	4,72	6,62	9,63	0,48
7	ChCl:U:Gly	1:2:2	DES 7.1	0,00	6,21	13,88	17,20	16,22	9,01	6,87	11,68	13,17	0,26
			DES 7.2	0,00	6,28	11,94	10,32	9,16	5,93	5,46	8,43	10,71	0,42
			DES 7.3	0,00	6,31	11,03	7,09	5,83	4,48	4,79	6,91	9,55	0,49
8	ChCl:EG	1:2	DES 8.1	0,00	3,88	10,32	24,30	20,33	6,54	6,31	10,36	13,56	0,22
			DES 8.2	0,00	5,12	10,17	13,89	11,23	4,70	5,18	7,79	10,91	0,40
			DES 8.3	0,00	5,70	10,10	8,97	6,93	3,83	4,65	6,57	9,66	0,48
9	ChCl:Gly	1:2	DES 9.1	0,00	5,67	11,81	22,50	21,63	8,31	7,00	12,63	13,64	0,25
			DES 9.2	0,00	6,03	10,86	12,38	11,25	5,41	5,43	8,68	10,78	0,42
			DES 9.3	0,00	6,19	10,45	8,04	6,80	4,17	4,76	6,99	9,55	0,50
10	ChCl:PG	2:3	DES 10.1	0,00	3,81	10,45	23,95	24,62	9,58	6,28	10,24	13,96	0,25
			DES 10.2	0,00	5,14	10,23	13,25	12,86	6,07	5,11	7,62	10,98	0,42
			DES 10.3	0,00	5,74	10,18	8,81	6,45	3,68	4,57	6,50	9,71	0,49

Abbreviations: betaine (B), choline chloride (ChCl), ethylene glycol (EG), glycerol (Gly), propylene glycol (PG), urea (U).

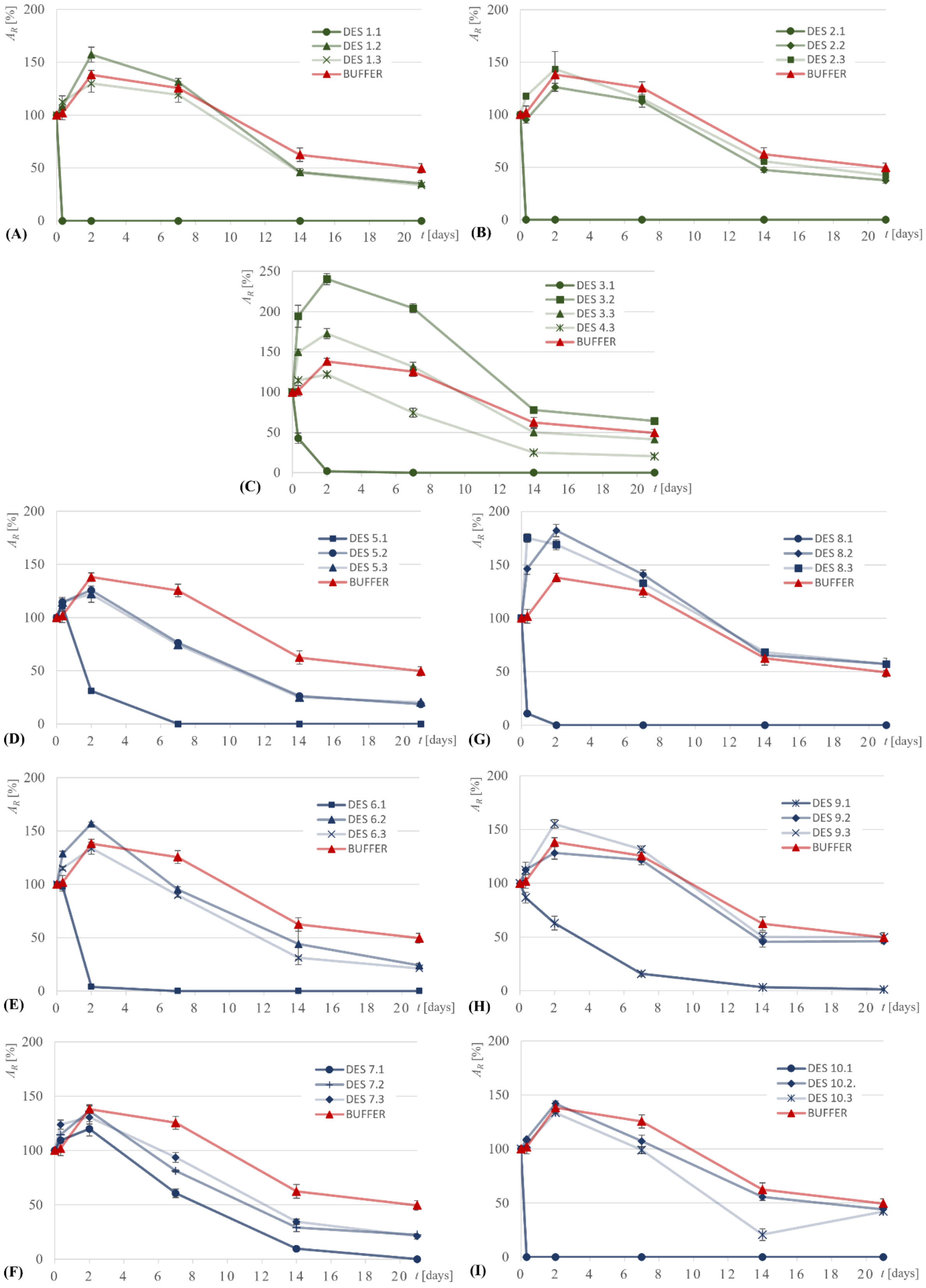

Supplementary Figure S1. Residual ADH-A activity (A_R in %) over time in different DES ((A-C) betaine-based in green and (D-I) choline chloride-based in blue) compared to 50 mM TRIS-HCl buffer, pH=7.5 (red)

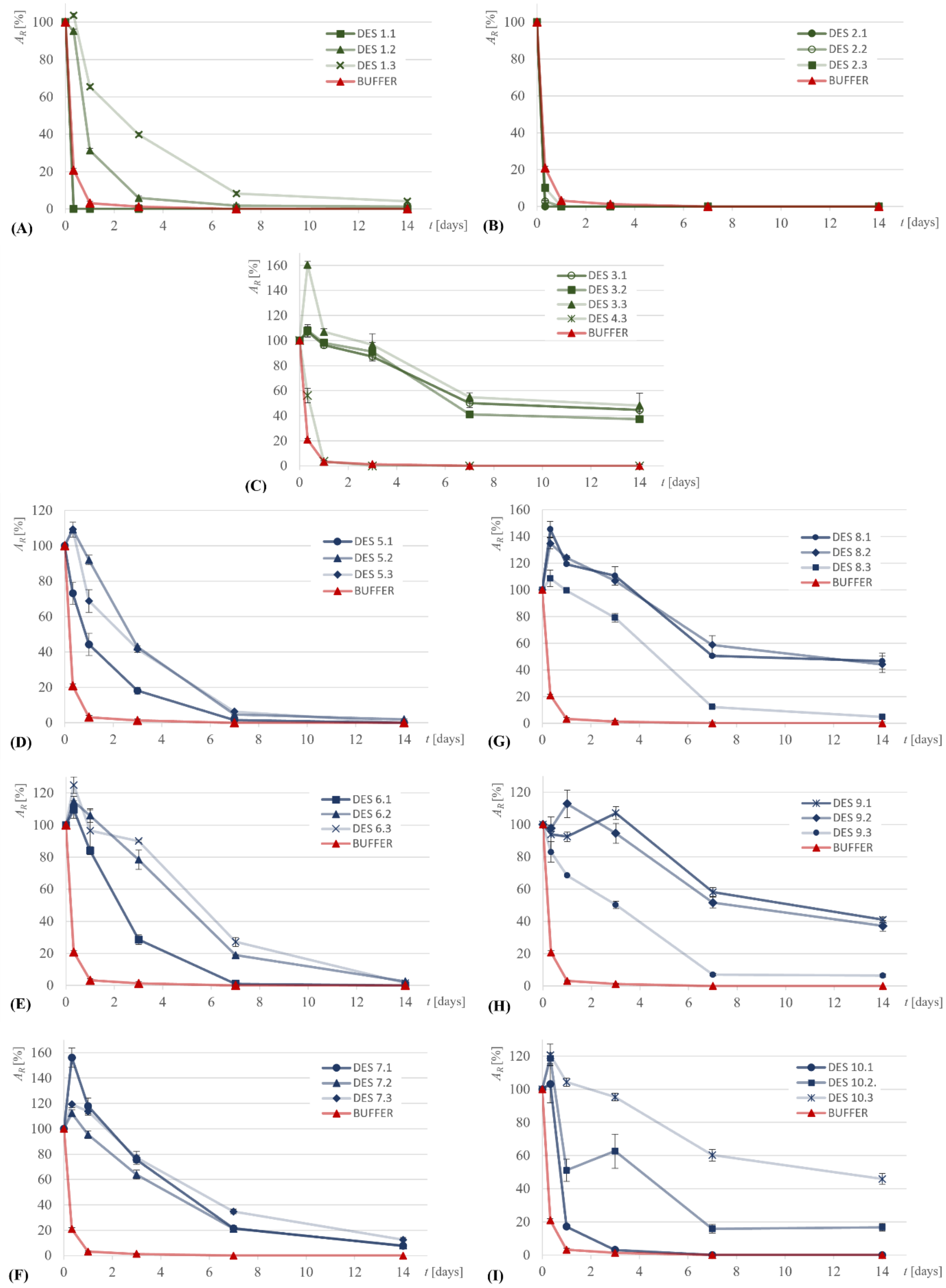

Supplementary Figure S2. Residual GDH activity (A_R in %) over time in different DES ((A-C) betaine-based in green and (D-I) choline chloride-based in blue) compared to 50 mM potassium-phosphate buffer, pH=7.5 (red)

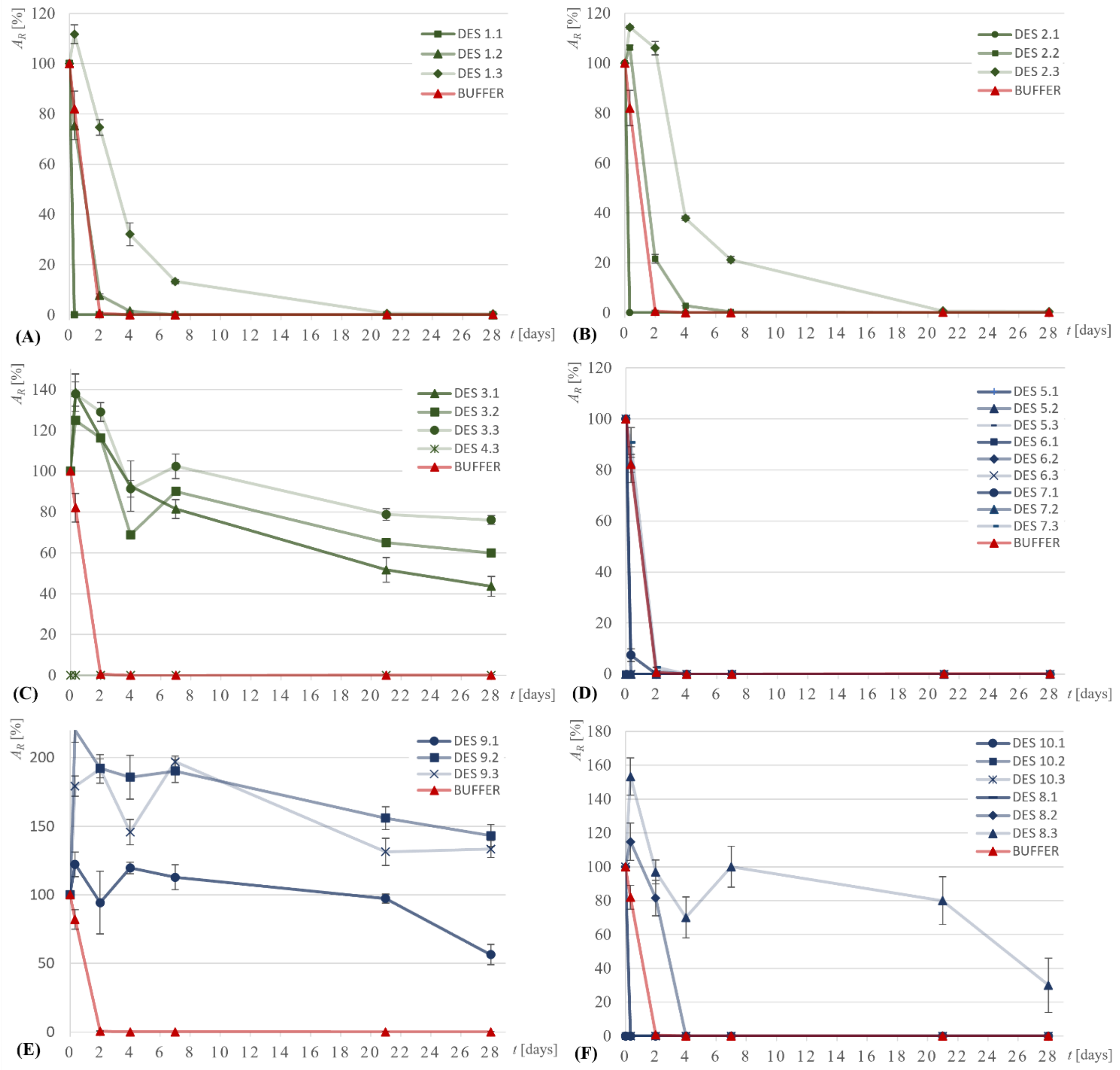

Supplementary Figure S3. Residual Lk-ADH activity (A_R, %) over time in different DES ((A-C) betaine-based in green and (D-F) choline chloride-based in blue) compared to 50 mM TRIS-HCl buffer, pH=7.5 (red)

Supplementary Table S2. The enzymes' inactivation rate constants (k, h^{-1}) in different DES and reference buffer.

	Abbrev. (HBA:HBD)	Molar ratio	DES code	ADH-A k (h^{-1}) ± st.dev.	ADH-A R^2	GDH k (h^{-1}) ± st.dev.	GDH R^2	Lk-ADH k (h^{-1}) ± st.dev.	Lk-ADH R^2
1	B:EG	1:2	DES 1.1	0.0816 ± 0.0147	0.7898	50.9799 ± 0.5304	0.9999	51.0000 ± 0.5301	0.9999
			DES 1.2	0.0008 ± 0.0001	0.8944	0.0309 ± 0.0025	0.9665	0.0399 ± 0.0017	0.9980
			DES 1.3	0.0010 ± 0.0002	0.9253	0.0135 ± 0.0007	0.9842	0.0101 ± 0.0049	0.9757
2	B:PG	1:3	DES 2.1	0.9639 ± 0.0228	0.9985	50.9799 ± 0.5304	0.9999	1.8737 ± 0.0000	0.9999
			DES 2.2	0.0017 ± 0.0002	0.9761	0.4397 ± 0.0891	0.9930	0.0279 ± 0.0162	0.9728
			DES 2.3	0.0013 ± 0.0002	0.9479	0.1711 ± 0.0843	0.9690	0.0079 ± 0.0005	0.9629
3	B:Gly	1:2	DES 3.1	0.0910 ± 0.0326	0.9851	0.0026 ± 0.0001	0.9937	0.0012 ± 0.0001	0.9680
			DES 3.2	0.0016 ± 0.0000	0.9290	0.0031 ± 0.0002	0.9897	0.0009 ± 0.0001	0.9710
			DES 3.3	0.0017 ± 0.0003	0.9311	0.0037 ± 0.0004	0.9679	0.0004 ± 0.0001	0.9751
4	B:U	1:3	DES 4.3	0.0026 ± 0.0001	0.9691	0.0722 ± 0.0339	0.9829		
5	ChCl:U	1:2	DES 5.1	0.0231 ± 0.0160	0.9632	0.0298 ± 0.0075	0.9951		
			DES 5.2	0.0021 ± 0.0002	0.9503	0.0111 ± 0.0007	0.9752		
			DES 5.3	0.0022 ± 0.0002	0.9645	0.0127 ± 0.0007	0.9807		
6	ChCl:U:EG	1:2:2	DES 6.1	0.0342 ± 0.0013	0.9702	0.0147 ± 0.0011	0.9652		
			DES 6.2	0.0022 ± 0.0003	0.9147	0.0076 ± 0.0004	0.9888	51.0000 ± 0.5301	0.9999
			DES 6.3	0.0024 ± 0.0002	0.9630	0.0070 ± 0.0004	0.9873	51.0000 ± 0.5301	0.9999
7	ChCl:U:Gly	1:2:2	DES 7.1	0.0037 ± 0.0003	0.9605	0.0083 ± 0.0006	0.9736	0.2325 ± 0.0688	0.9913
			DES 7.2	0.0026 ± 0.0003	0.9573	0.0083 ± 0.0024	0.9957	0.0450 ± 0.0209	0.9884
			DES 7.3	0.0025 ± 0.0002	0.9728	0.0059 ± 0.0003	0.9912	0.0401 ± 0.02305	0.9813
8	ChCl:EG	1:2	DES 8.1	0.1784 ± 0.0096	0.9589	0.0031 ± 0.0003	0.9789	51.0000 ± 0.5301	0.9999
			DES 8.2	0.0015 ± 0.0002	0.9449	0.0033 ± 0.0002	0.9876	0.0188 ± 0.0022	0.7916
			DES 8.3	0.0015 ± 0.0002	0.9449	0.0076 ± 0.0004	0.9868	0.0010 ± 0.0002	0.9205
9	ChCl:Gly	1:2	DES 9.1	0.0087 ± 0.0003	0.9942	0.0023 ± 0.0002	0.9878	0.0005 ± 0.0001	0.9700
			DES 9.2	0.0018 ± 0.0000	0.9703	0.0032 ± 0.0002	0.9932	0.0002 ± 0.0000	0.9590
			DES 9.3	0.0016 ± 0.0002	0.9519	0.0109 ± 0.0005	0.9881	0.0003 ± 0.0001	0.9572
10	ChCl:PG	2:3	DES 10.1	0.2778 ± 0.0011	0.9825	0.0498 ± 0.0046	0.9649	51.0000 ± 0.5301	0.9999
			DES 10.2	0.0016 ± 0.0002	0.9711	0.0097 ± 0.0008	0.9627	51.0000 ± 0.5301	0.9999
			DES 10.3	0.0014 ± 0.0002	0.9809	0.0030 ± 0.0001	0.9951	0.0031 ± 0.0013	0.5861
			Buffer*	0.0013 ± 0.0002	0.9659	0.1500 ± 0.0067	0.9836	0.0484 ± 0.0280	0.9814

Abbreviations: betaine (B), choline chloride (ChCl), ethylene glycol (EG), glycerol (Gly), propylene glycol (PG), urea (U).
(*) - 50mM TRIS-HCl, pH=7.5 for ADH-A and Lk-ADH; 50 mM potassium-phosphate buffer (pH=7.5) for GDH.
